# Design of Graphene- and Polyaniline-Containing Functional Polymer Hydrogel as a New Adsorbent for Removal of Chromium (VI) Ions

**DOI:** 10.3390/polym8120445

**Published:** 2016-12-21

**Authors:** Jae-Seo Chin, Anantha-Iyengar Gopalan, Nallal Muthuchamy, Kwang-Pill Lee

**Affiliations:** 1 Department of Chemistry Education, Kyungpook National University, Daegu 41566, Korea; dduksoon@chol.com (J.-S.C.); nm2020mnature@gmail.com (N.M); 2 Department of Nanoscience and Nanotechnology, Kyungpook National University, Daegu 41566, Korea; algopal99@gmail.com; 3 Research Institute of Advanced Energy Technology, Kyungpook National University, Daegu 41566, Korea

**Keywords:** functional gel, graphene, polyaniline, chromium ions

## Abstract

Hydrogels find applications in various fields, and the ever-growing spectrum of available monomers, crosslinking, and nanotechnologies widen the application of polymer hydrogels. Herein, we describe the preparation of a new graphene (G)- and polyaniline (PANI)-containing functional polymer gel (G/PANI/FG) through a facile crosslinking copolymerization approach. Several characterization techniques such as field-emission scanning electron microscopy, Fourier-transform infrared, and X-ray photoelectron spectroscopy were employed to understand the physicochemical characteristics of the G/PANI/FG. The new G/PANI/FG was used as an adsorbent for chromium (VI) and exhibited the highest Cr (VI) removal efficiency (~97%). The inclusion of G and PANI in the gel results in high surface area, 3D porous structure, and Cr (VI)-chelating amine sites, which enhanced the Cr (VI) removal efficiency and thermal stability of the gel adsorbent. The results of our study revealed that G/PANI/FG is suited for the removal of Cr (VI) from aqueous solution.

## 1. Introduction

Chromium (Cr), one of the most significant metal pollutants, is widely used by modern industries such as that of textiles and electroplating leather, and is likely to be discharged into the environment above the threshold limit. In natural waters, Cr exists mainly in two different oxidation states, Cr (VI) and Cr (III). The United States sets a maximum contamination for total Cr in drinking water of 100 µg/L, but a more stringent threshold for Cr is set as 50 µg/L by the World Health Organization (WHO). Cr (VI) is considered to be toxic because of its adverse effects on the liver, kidney, and lung functions [1]. There are differences in solubility, mobility, and toxicity between Cr (VI) and Cr (III) species. As compared with Cr (VI), Cr (III) is less soluble and mobile and can be precipitated as a hydroxide [2]. Besides, Cr (III) is nearly 1000 times less toxic than Cr (VI) and is also an essential nutrient for living organisms [3]. Different methods—such as precipitation, solvent extraction chemical and electrochemical techniques, ion-exchange methods, ultrafiltration and reverse osmosis, flotation, and coagulation—have been developed for the removal of Cr (VI) ions from industrial effluents and wastewaters [4,5,6,7]. However, most of these processes are unacceptable for practical Cr (VI) removal, owing to the difficulties in the disposal of sludge, high cost, low efficiency, and non-applicability to a wide range of conditions. As compared to other methods, adsorption is a well-known separation method and recognized as one of the efficient and economic methods for water decontamination applications. Also, owing to the reversible nature of most of the adsorption processes, the adsorbents can be regenerated by suitable desorption processes for multiple uses, and many desorption processes are of low maintenance cost while being highly efficient. It is to be noted that the recent trend in Cr (VI) removal strategies involves the reduction of Cr (VI) to Cr (III) followed by adsorption [8,9,10]. Also, the development of novel adsorbents, which can concomitantly perform adsorption and reduction of Cr (VI) in situ, has proved to be an effective approach for the removal of Cr (VI) from the aquatic system [11,12]. Therefore, there is a need to develop new effective adsorbents which can support both adsorption of Cr (VI) and transformation of Cr (VI) to Cr (III). Taking these aspects into account, the present study focuses on the development of a new kind of graphene (G)- and polyaniline (PANI)-containing functional gel adsorbent.

Some adsorbents, such as activated carbon, zeolites, clays, and agricultural residues, have been widely researched for Cr (VI) removal. However, the disadvantages of these adsorbents include low adsorption capacities, weak interactions with the metallic (pollutant) ions, and have difficulties performing separation and regeneration. Ion-exchange resins have also been developed to remove metal ions; however, they have low selectivity and show a high degree of swelling combined with poor mechanical stability. Gels, based on organic polymers, have proved to be the most promising adsorbents, as they can be incorporated with functional groups to have adequate interactions with the metal ions [13,14,15]. Gels containing function groups such as –OH and –C=O showed excellent adsorption properties for the removal of Cr (VI) [16]. However, many of the gel adsorbents did not meet the practical requirements of good adsorption capacity and selectivity towards Cr (VI) and subsequent reduction to lesser toxic Cr (III) species.

PANI is known to exist in different oxidation states, including emeraldine base (EB), leucoemeraldine base (LEB), and pernigraniline base (PB). The oxidation states in PANI are interconvertible and can be exploited for the effective removal of Cr (VI) [12,17,18]. PANI can be prepared in various physical states—such as powder, film, and different nanostructured forms—and utilized for Cr (VI) removal [19,20]. Having understood the problems associated with pristine PANI for Cr (VI) removal, sawdust-coated PANI [21]; nylon composite [22]; and polyacrylonitrile/PANI core–shell nanofibers [23] have been developed. Nanosized carbon materials, such as G, are adsorbents that are of great importance because of their high surface area and good adsorption capacities [24]. G/Fe nanocomposites [25], G/Fe_3_O_4_ nanocrystals [10], and G/TiO_2_ nanocomposites [26] have been used for Cr (VI) removal.

**Scheme 1:**
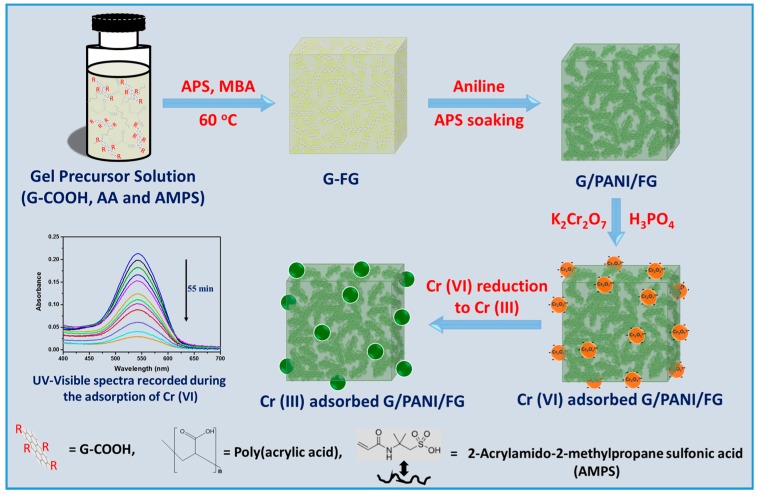
Scheme showing the preparation of graphene (G)- and polyaniline (PANI)-containing functional gel (G/PANI/FG) and removal of Cr (VI). AA: acrylic acid; APS: ammonium persulfate; MBA: *N*,*N*′-methylenebisacrylamide.

Keeping the above aspects in view, we designed and synthesized a new kind of G- and PANI-containing gel adsorbent for Cr (VI) removal (Scheme 1). G provides the high surface area for good Cr (VI) adsorption, and the structural properties of PANI support the electron transfer with Cr (VI) to mediate the conversion of carcinogenic Cr (VI) to nontoxic Cr (III). Our results demonstrated the high potential for removal and detoxification of Cr (VI) in an aqueous medium using the newly developed G- and PANI-containing functional gel (designated as G/PANI/FG), where the parent gel was composed of poly(acrylamido-2-methylpropane sulfonic acid-*co*-acrylic acid), namely P(AMPS-*co*-AA).

## 2. Materials and Methods

### 2.1. Materials

*N*,*N*′-methylenebisacrylamide (MBA), ammonium persulfate (APS), acrylic acid (AA), 2-acrylamido-2-methylpropane sulfonic acid (AMPS), and diphenyl carbazide (DPC) were obtained from Sigma-Aldrich (Seoul, Korea). G nanoplatelet sheets were obtained from E-Nano TEC (Kwanju, Korea). Aniline and potassium dichromate (K_2_Cr_2_O_7_) were purchased from Duksan reagents (Ansan, Korea). Phosphoric acid (H_3_PO_4_), hydrochloric acid (HCl), and other reagents used in this work were analytical reagent grade samples. Distilled water was employed exclusively in our experiments.

### 2.2. Preparation of G/PANI/FG

G/PANI/FG was prepared using the parent gel based on P(AMPS-*co*-AA), which was prepared using the two acrylic monomers, AMPS and AA along with MBA as the crosslinker (Scheme 1). Typically, the preparation procedure involved two major stages. In stage 1, the G-containing P(AMPS-*co*-AA) gel (G/P(AMPS-*co*-AA)/gel) was prepared, and in the second stage, G/P-AMPS-*co*-AA/gel was further combined with PANI to obtain G/PANI/FG1.

#### 2.2.1. Preparation of G/P(AMPS-*co*-AA)

G sheets were functionalized with COOH (G–COOH) groups as detailed elsewhere [27]. About 10 mg of G–COOH was dispersed in 35 mL of aqueous solution and stirred under sonication for 1 h. Subsequently, AMPS (0.125 M), AA (1.125 M), and MBA (5 mM) were included in the G suspension and stirred for an additional 30 min. The solution mixture was purged with N_2_ gas to remove the dissolved oxygen, and heated to 60 °C. About 5 mL of 0.2 M APS was added dropwise for 30 min into the mixture (kept at 60 °C) and stirred for 6 h to obtain G/P(AMPS-*co*-AA)/gel. For comparative purposes, three other gels were prepared following a similar procedure adopted for G/P(AMPS-*co*-AA)/gel, but in the absence of either AA, AMPS, or G (Table 1).

#### 2.2.2. PANI Modification on G/P(AMPS-*co*-AA)/PANI/FG

PANI was included within the above-prepared gels, and the PANI modified gels, G/PANI/FG1, G/PANI/FG2, G/PANI/FG3, and PANI/GNG (Table 1) were prepared through polymerization of aniline within the respective precursor gel matrix. Typically, the prepared precursor gel was put in a solution containing 50 mM of aniline in 1 M HCl (20 mL) and well stirred for 30 min at 5 °C. Further, about 5 mL of 0.2 M APS was added dropwise for 30 min and the mixture was maintained for 6 h. The color of the parent gel changed into green. The PANI-included gels (designated in Table 1) were removed from the solution, washed with 0.1 M HCl, and stored in 0.1 M HCl.

### 2.3. Instrumentation

Field-emission scanning electron microscope (FE-SEM) (model S-4300 from Hitachi, Tokyo, Japan) was used to observe the morphology of the hydrogels. First, the hydrogels were allowed swell to reach equilibrium in distilled water at room temperature, and then exchanged by ethanol to obtain dried gels. Finally, the samples were sputter-coated onto the gold surface to perform FE-SEM. Fourier-transform infrared (FTIR) spectra were recorded using Bruker IFS 66v FT-IR spectrophotometer (BRUKER OPTIK, Billerica, MA, USA) in the wavenumber range 400–4000 cm^−1^. Thermogravimetric analysis was performed at a scanning rate of 10 °C·min^−1^ using a thermogravimetric analyzer (Q600, TA Instrument, New Castle, DE, USA) with samples placed in a nitrogen atmosphere (temperature range from 30 to 800 °C). UV–visible spectra were collected using a Cary 5000, Agilent (Santa Clara, CA, USA) UV/vis/NIR spectrometer. X-ray photoelectron spectroscopy (XPS) (Quantera SXM from ULVAC-PHI, Kanagava, Japan) was used for the adsorbent surface analysis both before and after Cr (VI) treatment. Nitrogen adsorption–desorption was performed (Autosorb-iQ-Quadrasorb SI, Kanagava, Japan) using Brunauer–Emmet–Teller (BET) approach to obtain the pore properties of the gels.

### 2.4. Experiments for Removal of Cr (VI) 

In a typical batch experiment, 20 mg of G/PANI/FG1 was added to a 20 mL solution of 0.2 M H_3_PO_4_ (pH adjusted to 2.0) containing K_2_Cr_2_O_7_ (6 mg·L^−1^). The Cr (VI) adsorption capability of the gel was monitored as follows. About 0.1 mL of Cr (VI) solution was withdrawn from the mixture at various time intervals, diluted properly with 0.2 M H_3_PO_4_, and used for recording the UV–visible spectra. The solutions collected in this manner were used for the determination of residual Cr (VI) utilizing Cr (VI)–DPC complex chemistry. Specifically, 0.5 mL of the Cr (VI) solution was mixed with 0.12 mL of DPC (20 mg/5 mL acetone) and 2.8 mL of 0.2 M H_3_PO_4_. The solution was allowed to stand for 5 min to obtain a stable purple color. The Cr (VI) concentration was determined by monitoring the absorbance at 550 nm. The Cr (VI) determination experiments were repeated thrice. The Cr (VI) concentrations were deduced from the average of absorbances at 550 nm. The Cr (VI) removal percentage (*R*%) and the removal capacity (*Q*) were calculated from the absorbance values at 550 nm. *R*% was calculated using the equation:

(1) $$R\%=\frac{C_{o}-C_{e}}{C_{o}}\times 100$$

where *C*_o_ and *C*_e_ are the initial and final Cr (VI) ion concentration (mg·L^−1^), respectively, after adsorption into the respective gel. *Q* (mg·g^−1^) of the gel was obtained through the Equation (2).

(2) $$Q=\frac{(C_{o}-C_{e})V}{m}$$

in which *V* is the volume of the Cr (VI) ion solution (mL) and *m* is the mass of gel in g.

### 2.5. Reproducibility

The reproducibility of gel preparation and Cr (VI) adsorption was evaluated as follows. Typically, G/PANI/FG1 was selected for the evaluation of reproducibility. The synthesis procedures in stage 1 and stage 2 were identically triplicated and the final products were collected from the batch experiments. Besides, the G/PANI/FG1 adsorbent prepared in a particular batch was fractionated into three parts, and the Cr (VI) removal experiment was individually carried out using the fractionated samples. Thus, reproducibility was evaluated for the three batch-prepared G/PANI/FG1 samples and three fractionated samples from a particular batch with respect to Cr (VI) removal efficiency under similar conditions of adsorption experiments. The relative standard deviation of Cr (VI) removal efficiency was less than 2.3%, suggesting reproducibility of the gel and Cr (VI) adsorption measurements.

## 3. Results and Discussion

### 3.1. Morphology

**Figure 1:**
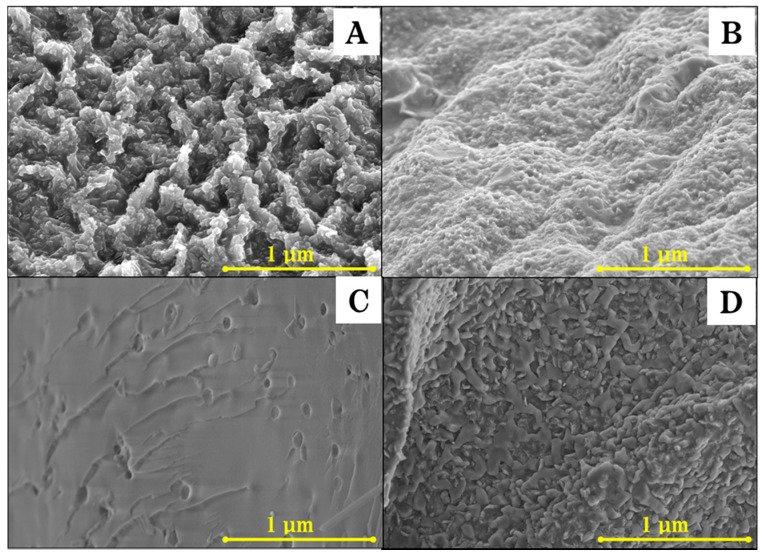
Field-emission scanning electron microscope (FE-SEM) images of (**A**) G/PANI/FG1; (**B**) G/PANI/FG2; (**C**) G/PANI/FG3; and (**D**) PANI/GNG.

The G- and PANI-containing gels were prepared using the protocol described in Section 2.2. The morphology of the gels was observed by FE-SEM (Figure 1). The G- and PANI-containing gels, G/PANI/FG1, G/PANI/FG2, and G/PANI/FG3 show three-dimensional (3D) porous structures (Figure 1A–C). Careful observation of the FE-SEM images reveals the difference between the morphology of G- and PANI-containing gels (Figure 1A–C) and PANI/GNG (Figure 1D). The surface of the G/PANI/FG1 is rugged, rougher, and more porous in nature as compared to the other gels. Typically, G/PANI/FG1 possesses interconnected large pores with thicker walls (Figure 1A) as compared to G/PANI/FG2 (Figure 1B) and G/PANI/FG3 (Figure 1C). However, PANI/GNG, where G was not included gel, shows a rougher surface containing a larger number of shorter wormlike materials (Figure 1D). Figure 1 shows variations in the surface roughness amongst the prepared gels. The variations in the surface roughness are attributed to the difference in the polymeric backbones which constitute the respective gel (Table 1, Column 2). Typically, the difference in surface morphology between G/PANI/FG1 (Figure 1A) and PANI/GNG (Figure 1D) originates from the inclusion of G. In all, the G- and PANI-containing gels contain G sheets with an interconnected polymer network as the crosslinker to produce 3D porous gels. In our work, we used G sheets functionalized with –COOH groups, which enabled the formation of 3D composite gels through crosslinking interactions between functionalized G sheets and the polymer chains, via either covalent bonds or noncovalent (hydrogen bond) interactions [28,29]. We synthesized G/PANI/FG1, G/PANI/FG2, G/PANI/FG3, and PANI/GNG (Table 1) through two stages. In the first stage, the respective precursor gel was prepared (Table 1, Column 2). The synthetic conditions to prepare the gels caused linking of rigid organic units, to impart rigidity within the network and further provide directionality for the formation of the extended network. The rigidity prevents the collapse of the network upon itself and results in free volume, which becomes the pores within a framework. The combination of AA and other gel-forming monomers such as MBA and AMPS results in interconnected polymer chain networks, which in turn generate macro/mesopores. The pores or voids in the gels can subsequently be encapsulated or partially filled with another polymer (PANI, in our case). Such pore-filled hydrogels demonstrated outstanding performance for separation of ions [30,31]. In this work, the precursor gels were subsequently loaded with PANI to enable effective Cr (VI) removal. Necessarily, the macro/mesopores in the precursor gels may be filled with PANI in the second stage of our synthetic procedure. The internal pore morphology of the gels was characterized by the change in porosity after filling the precursor gel with PANI. We determined the pore sizes of the G/PANI/FG1, G/PANI/FG2, G/PANI/FG3, and PANI/GNG by nitrogen adsorption–desorption measurements. The average pore radius of G/PANI/FG1 and PANI/GNG were 1.50 and 2.14 nm, respectively. Thus, the inclusion of G in the gel caused a decrease in pore radius. The gels G/PANI/FG2 and G/PANI/FG3 (Table 1, Column 2), prepared with the absence of one of the comonomer (AMPS or AA), exhibited much larger pore radii of 3.70 and 2.06 nm, respectively, as compared to the pore radius of G/PANI/FG1 (1.50 nm). It is observed that G/PANI/FG1 has the lowest pore radius amongst the four gels and hence expected to be filled with a larger amount of PANI. The specific surface area of the gels takes the order G/PANI/FG1 > G/PANI/FG2 > PANI/GNG > G/PANI/FG3.

### 3.2. FTIR Studies

**Figure 2:**
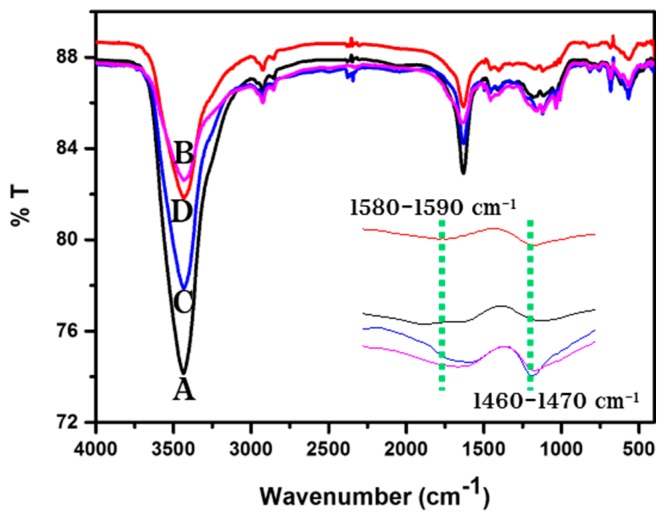
Fourier-transform infrared (FTIR) spectrum of (**A**) G/PANI/FG1; (**B**) G/PANI/FG2; (**C**) G/PANI/FG3; and (**D**) PANI/GNG.

Figure 2 shows the FTIR spectrum of the G/PANI/FG1, G/PANI/FG2, G/PANI/FG3, and PANI/GNG. The wavenumbers corresponding to a few of the absorption bands are indicated in Figure 2. The broad band around 3430 cm^−1^ is attributed to O–H stretching vibration of –COOH group in polyacrylic acid (PAA), N–H stretching vibration of the –CONH_2_ group in polyAMPs (PAMPS), and also from O–H stretching vibration of the –SO_3_H groups in PAMPS. Due to the chemical structures of the gels, the occurrence of hydrogen bonding is possible between –SO_3_H groups and –CONH_2_ groups and/or involving the –COOH groups in PAA and G–COOH. The C=O stretching band of AMPS (amide-I) is observed around 1635–1650 cm^−1^. The stretching vibrations of the quinoid and benzenoid rings of PANI are observed around 1580–1590 cm^−1^ and 1460–1470 cm^−1^, respectively [32]. The benzenoid-stretching peaks are found to be more intense and sharper as compared to the intensities of benzenoid-stretching ring peaks of PANI. There can also be interactions between the PANI chains and the π-bonded graphene within the hydrogel [33]. The quinoid PANI peak (~1500 cm^−1^) is weaker, which can be rationalized because of the restricted modes of vibrations due to the tightly packed PANI particles between the G–COOH layers.

### 3.3. Thermogravimetric Analysis (TGA)

**Figure 3:**
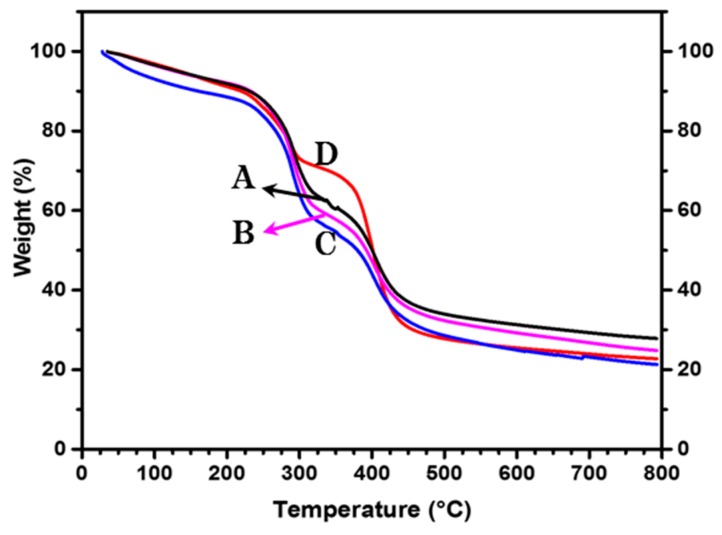
Thermogravimetric analysis (TGA) curves of (**A**) G/PANI/FG1; (**B**) G/PANI/FG2; (**C**) G/PANI/FG3; and (**D**) PANI/GNG.

Thermal properties of the various gels—G/PANI/FG1, G/PANI/FG2, G/PANI/FG3, and PANI/GNG—were investigated and the TGA curves are presented in Figure 3A–D. The first stage thermal degradation (of about 15% weight loss) was observed between 30 and 170 °C, which arises due to the elimination of moisture in the gels. The second stage weight loss from 170 to 300 °C may be due to the degradation of –COOH groups in the gels. The gel PANI/GNG (Figure 3D) showed lower weight loss % up to 300 °C as compared to the other gels (Figure 3A–C). This is due to the fact that the gels, excepting PANI/GNG, contain G–COOH. The degradation of –COOH groups in G–COOH caused higher weight losses for the three other gels. The third stage weight loss in the range 300–450 °C can be attributed to thermal degradation of –SO_3_H groups. The –SO_3_H groups are thermally more stable than –COOH groups [34]. The second and third stage weight losses together contributed to nearly 50%–60%, which indicated higher content of the gel polymers PAA and PAMPS in the respective gels. The thermal stability of gels was ascertained by comparing the weight loss % in the temperature range from 300 to 450 °C (the temperature range at which the polymer backbone structure of the gels degrades). G/PANI/FG1, G/PANI/FG2, G/PANI/FG3, and PANI/GNG exhibited weight losses of 32.7%, 35.2%, 33.7% and 43.4%, respectively. We specifically compared the thermal stability of G/PANI/FG1 and PANI/GNG, the two gels which were prepared from the two monomers AA and AMPS (Table 1, Rows 1 and 4). G/PANI/FG1 contains G–COOH, whilst PANI/GNG does not include of G–COOH. The weight loss % of G/PANI/FG1 and PANI/GNG were 32.7% and 43.4%, respectively, in the temperature range between 300 and 450 °C. Clearly, the G–COOH-containing gel (G/PANI/FG1) exhibited lower weight loss % and hence more thermally stable. Thus, we conclude that inclusion of G–COOH caused better thermal stability for the gel. The final stage weight loss of the gels was observed from 450 to 700 °C, which is due to the degradation of PANI chains. Importantly, the gel without G, PANI/GNG, showed a larger weight change between 300 and 450 °C, as compared to the G-containing gels, G/PANI/FG1, G/PANI/FG2, and G/PANI/FG3. Thus, TGA results in Figure 3A–D revealed that the extent of degradation of the polymer chains decreased by the inclusion of G, which suggested improvement in thermal stability for the G-containing gels, G/PANI/FG1, G/PANI/FG2, and G/PANI/FG3.

### 3.4. Adsorption Kinetics of Cr (VI)

**Figure 4:**
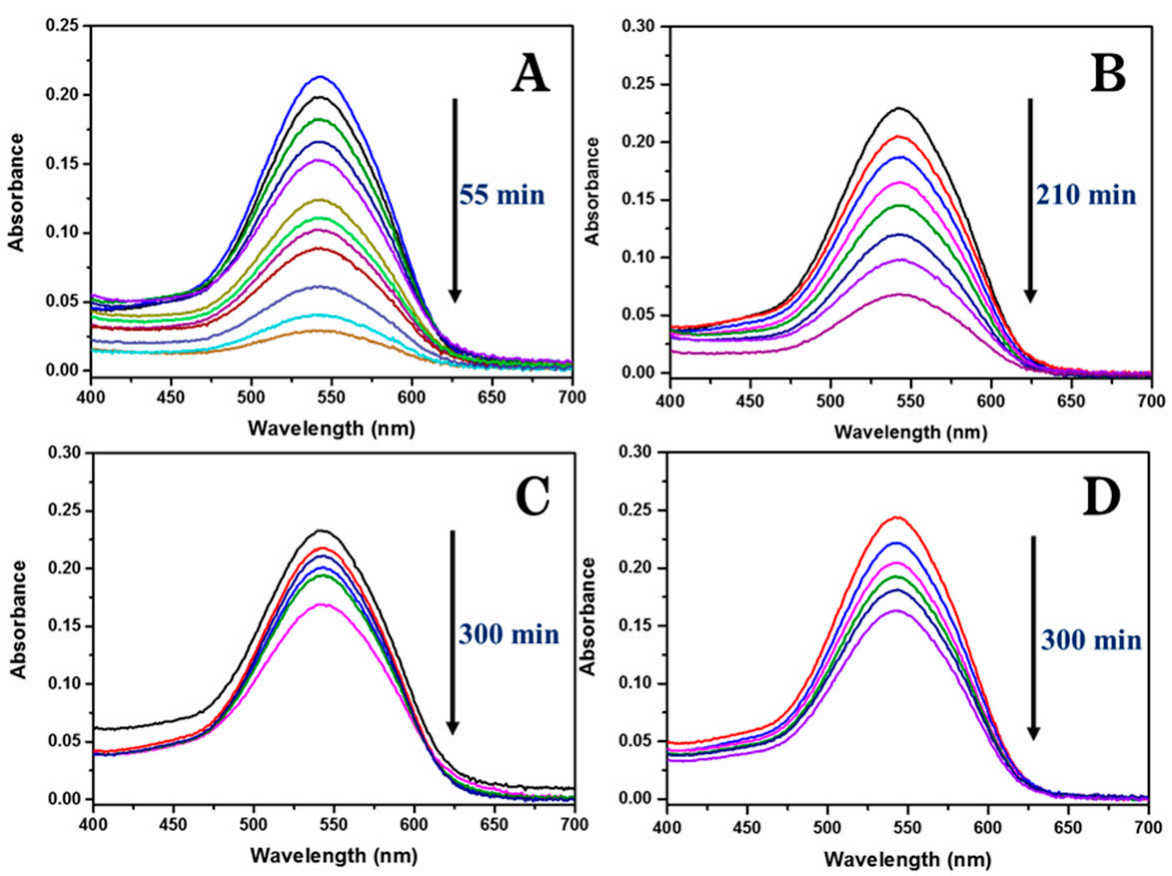
UV–visible spectra recorded during the adsorption of Cr (VI) in the presence of (**A**) G/PANI/FG1; (**B**) G/PANI/FG2; (**C**) G/PANI/FG3; and (**D**) PANI/GNG adsorbent (20 mg), Cr (VI) = 0.5 mM, pH = 2, and temperature = 25 °C.

**Figure 5:**
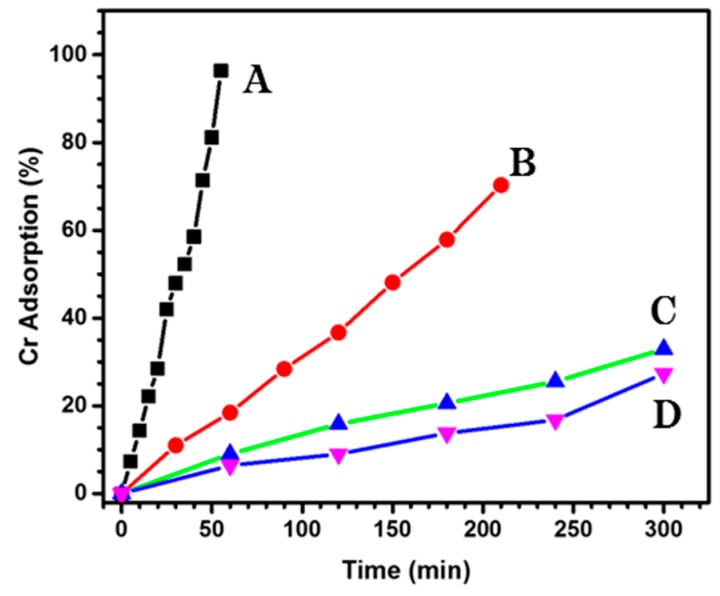
Effect of contact time on Cr (VI) removal (%) using (**A**) G/PANI/FG1; (**B**) G/PANI/FG2; (**C**) G/PANI/FG3; and (**D**) PANI/GNG on adsorption % of Cr (VI). Cr (VI) = 0.5 mM, pH = 2, and temperature = 25 °C.

The hexavalent chromium Cr (VI) exists as CrO_4_^2−^, HCrO_4_^−^, or Cr2O_7_^2−^, depending on the pH of the medium [35]. We studied the effect of pH on the removal of Cr (VI) using the prepared adsorbents. The pH of the Cr (VI) solution was adjusted using HCl or NaOH. The highest *Q* value was found in the pH range of 1–3, whereas the *Q* values were low both before and after the pH range 1–3. We considered the following reason for the pH effect on Cr (VI) removal. The adsorbents G/PANI/FG1, G/PANI/FG2, G/PANI/FG3, and PANI/GNG contain PANI as the functional polymer to augment the Cr (VI) removal. PANI generally exists in the protonated emeraldine salt form in the pH range 1–3 [36]. The color of the adsorbent was green in the pH range 1–3 and blue when the pH was above or below the pH range 1–3. The green color signifies the existence of emeraldine salt form of PANI in the pH range 1–3. The positively charged nitrogen in the emeraldine salt form of PANI chains electrostatically binds the negatively charged Cr (VI) (Cr_2_O_7_^2−^) ions in the pH range 1–3. Considering the maximum *Q* at pH = 2, we performed the subsequent Cr (VI) removal experiments at pH 2. Figure 4 presented the UV–visible spectrum recorded during the adsorption of Cr (VI) with different gels over the various contact period during adsorption. The time at which the adsorbate (Cr (VI)) in contact with the adsorbent (gel) that achieves the highest *Q* is considered as the measure of the efficiency of the adsorption by the adsorbent gel. Figure 5 depicts the effect of adsorption time on the *R*% of Cr (VI) for the various gels. Among the hydrogels, G/PANI/FG1 showed the highest *R*% value of 97% at 55 min (Figure 5A). The other gels—G/PANI/FG2, G/PANI/FG3, and PANI/GNG—exhibited *R*% of 70.3% (210 min), 28% (300 min), and 33% (300 min), respectively. The *Q* value of the gels was compared at 55 min and found to take the order: G/PANI/FG1 (97%) > G/PANI/FG2 (19%) > PANI/GNG (8%) > G/PANI/FG3 (6.3%). Importantly, the lowest *Q* was observed in PANI/GNG, which signifies the importance of G inclusion in the gel to achieve higher Cr (VI) removal. The *R*% of Cr (VI) removal and *Q* are influenced by the extent of pores by PANI. Specifically, results from the BET analysis revealed that PANI-loading is high with G/PANI/FG1 (as evident form the smallest pore radius among the gels) and the gel has higher surface area. These features caused G/PANI/FG1 to have higher *R*% and *Q*. The adsorption capacity of G/PANI/FG1, the best adsorbent from our results, is 300 mg/g, which is comparatively higher than reported for polyacrylonitrile/FeCl_2_ composite porous nanofibers [37], wood sawdust-coated polypyrrole [38], and polypyrrole/Fe_3_O_4_ nanocomposite [39]. The Cr (VI) adsorption % was monitored for the gel after regeneration of the gel by desorption.

**Figure 6:**
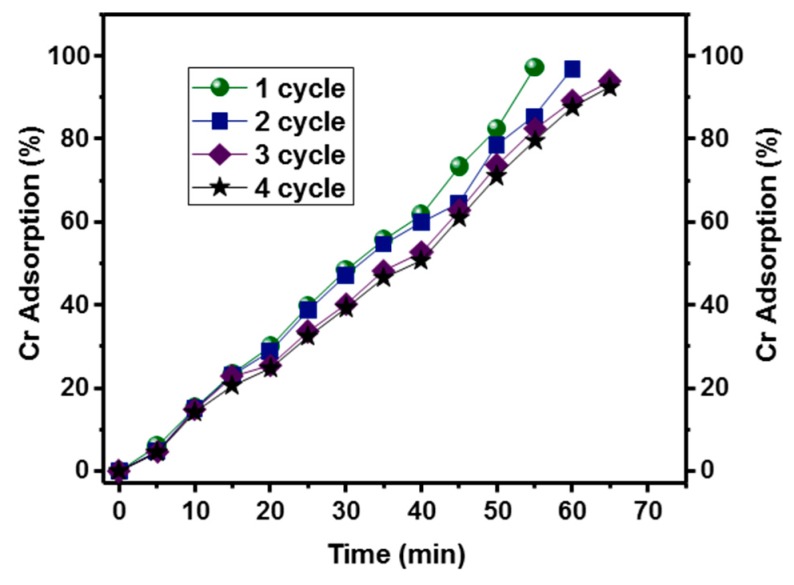
Reusability of G/PANI/FG1 for the adsorption of Cr (VI). Cr (VI) = 0.5 mM, pH = 2, and temperature = 25 °C.

The reusability of G/PANI/FG1 over repeated adsorption–desorption cycles was investigated. We earlier observed that the adsorption of Cr (VI) onto G/PANI/FG1 was pH-dependent and that pH 2 was optimal for the maximum Cr (VI) removal. The desorption of Cr species from the gel was done by keeping the pH above 3. The Cr (VI) adsorption % observed for the gel after each one of the adsorption–regeneration cycles is presented in Figure 6. The G/PANI/FG1 adsorbent exhibited *Q* of 92.5% after four adsorption–desorption cycles, meaning a decrease of 7.5% after four successive operations (Figure 6). The reason for the decrease in *Q* after four cycles may be the partial leaching of PANI during the desorption process at the alkaline condition.

### 3.5. Mechanism of Cr (VI) Adsorption

**Figure 7:**
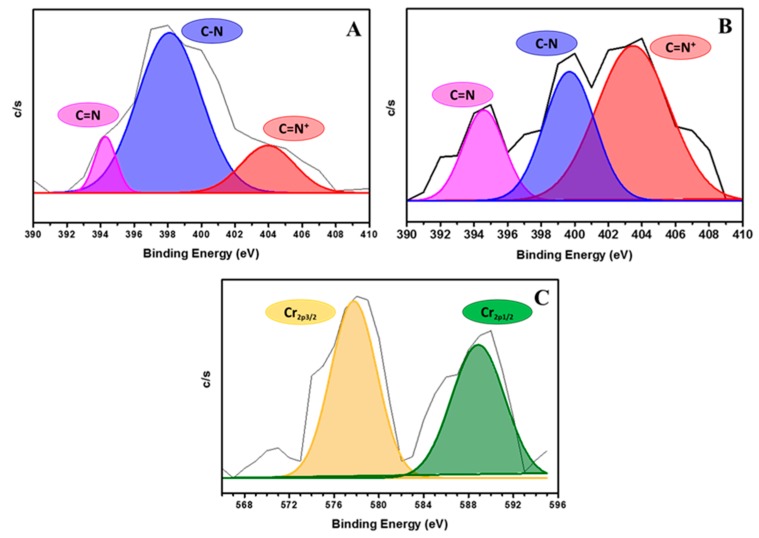
Deconvoluted N1s core-level X-ray photoelectron spectroscopy (XPS) spectrum of G/PANI/FG1 (**A**) before and (**B**) after Cr (VI) adsorption; (**C**) deconvoluted Cr_2p_ XPS spectrum of G/PANI/FG1 (after Cr (VI) adsorption).

To investigate the mechanism of Cr (VI) adsorption by G/PANI/FG1, high-resolution XPS spectra of N1s and Cr2p were used (Figure 7). The N1s core-level XPS spectrum of G/PANI/FG1, recorded before and after adsorption of Cr (VI), was deconvoluted into three kinds of nitrogen species (Figure 7A,B). The three components are assigned for C=N (394.63 eV), C–N (399.67 eV), and C=N^+^ (403.46 eV) based on the binding energy values. Figure 7A,B and Table 2 present the proportions of quinoid imine (C=N and C=N^+^) (Q) and benzoid amine (C–N) (B) units in the PANI chains. On close perusal of Table 2, it is clear that the ratio of Q (oxidized form) to B (reduced form) in PANI chains significantly increased from 0.366 before Cr (VI) adsorption to 6.27 after Cr (VI) adsorption. The increase in imine proportion in the G/PANI/FG1 after Cr (VI) adsorption signifies that PANI chains in G/PANI/FG1 are converted from reduced (–NH–) form to oxidized (=N– and =N^+^) form. Concurrently, Cr (VI) is expected to be reduced to Cr (III) state. The XPS scans of Cr_2p_ region (Figure 7C) shows two peaks with binding energy values 588.61 and 578.0 eV for Cr_2p1/2_ and Cr_2p3/2_ states, respectively. The magnitude of Cr_2p_ spin-orbit splitting is 10.61 eV, which is in accordance with the Cr (III) state [40,41]. Our XPS results confirmed that Cr (VI) is reduced to Cr (III) state (Figure 7C). 

## 4. Conclusions

In this study, a new graphene (G)- and polyaniline (PANI)-containing gel was prepared and utilized towards effective adsorption of Cr (VI) ions via amine chelation and higher adsorption features. The highest Cr (VI) removal (~97%) was witnessed for the gel prepared through the crosslinking reaction between of polyacrylic acid (PAA) and poly(2-acrylamido-2-methylpropane sulfonic acid) (PAMPS). The special features such as the larger amount of PANI- loading within the pores, the efficient chelating effect of amine groups towards Cr (VI), and transformation of Cr (VI) to Cr (III) contributed to the highest Cr (VI) removal by the gel. In conclusion, the data presented in this work suggested that the functional gel comprising G, PAA, PAMPS, and PANI is very suitable for efficient removal of Cr (VI) ions from its aqueous solutions.

## Figures and Tables

**Table 1:** Preparation of gels and designation.

Feed composition of the gel in stage I	Precursor gel	Further inclusion stage II	Final gel designation
G (10 mg), AA (1.125 M) and AMPS (0.125 M)	G/P(AMPS-*co*-AA)	PANI	G/PANI/FG1
G (10 mg) and AA (1.125 M)	G/* PAA	PANI	G/PANI/FG2
G (10 mg) and AMPS (0.125 M)	G/** PAMPS	PANI	G/PANI/FG3
AA (1.125 M) and AMPS (0.125 M)	P(AMPS-*co*-AA)	PANI	PANI/*** GNG

* Polyacrylic acid, ** Poly(2-acrylamido-2-methylpropane sulfonic acid), *** Graphene not included gel.

**Table 2:** The peak area % from XPS spectra.

	Area (%)	
	Before Cr (VI) adsorption	After Cr (VI) adsorption
C=N^+^	18.09	52.23
C–N	73.25	30.34
C=N	08.65	17.41
* imine/amine ratio	0.365	6.27

* Imine total (C=N + C=N^+^).
